# Anterior and Posterior Ocular Biometry in Healthy Chinese Subjects: Data Based on AS-OCT and SS-OCT

**DOI:** 10.1371/journal.pone.0121740

**Published:** 2015-03-23

**Authors:** Wenbin Huang, Xinbo Gao, Xingyi Li, Jiawei Wang, Shida Chen, Wei Wang, Shaolin Du, Mingguang He, Xiulan Zhang

**Affiliations:** Zhongshan Ophthalmic Center, State Key Laboratory of Ophthalmology, Sun Yat-Sen University, Guangzhou, People’s Republic of China; Massachusetts Eye & Ear Infirmary, Harvard Medical School, UNITED STATES

## Abstract

**Background:**

To measure the anterior and posterior ocular biometric characteristics concurrently and to determine the relationship between the iris and choroid in healthy Chinese subjects.

**Methods:**

A total of 148 subjects (270 eyes) were enrolled in this cross-section study. The anterior and posterior ocular biometric characteristics were measured simultaneously by anterior segment optical coherence tomography (AS-OCT) and swept-source optical coherence tomography (SS-OCT).

**Results:**

Compared with male eyes, female eyes had narrower anterior biometric parameters that presented with smaller anterior segment parameters [including anterior chamber depth (ACD), width (ACW), area (ACA), and volume (ACV); (all p<0.001)], narrower anterior chamber angle parameters [including angle opening distance (AOD750), trabecular–iris space area (TISA750), and angle recess area (ARA); (all p<0.001)], higher iris curvature (ICURV) (p = 0.003), and larger lens vaults (LV) (p = 0.019). These anterior ocular biometric parameters were correlated with increasing age (p<0.01). Iris thickness (IT750) and iris area (IAREA) were associated with age, ACW, and pupil diameter (all p<0.05), while choroidal thickness (CT) was associated with age, gender, and axial length (all p<0.05). Univariate regression analysis showed that greater CT was significantly associated with smaller IAREA (p = 0.026).

**Conclusion:**

Compared with male eyes, female eyes had narrower anterior biometric parameters that correlated with increasing age, which would be helpful in explaining the higher prevalence of angle closure rates in the female gender and in aging people. Increased CT might be associated with smaller iris area; however, this possibility needs to be investigated in future studies before this conclusion is made.

## Introduction

Ocular biometry is essential for understanding ocular growth and the development of ocular pathologies. Changes in of the anatomic structures of the eye may cause visual abnormalities, such as primary angle closure disease (PACD). The biometric characteristic of narrow angles remains one of the most important risk factors for PACD, and its modulation has been shown to influence both glaucoma onset and progression.

Many published studies have examined ocular biometry in PACD and have provided significant data. Previous studies found that PACD is characterized by biometric factors such as shallow anterior chamber depth (ACD), thick crystalline lens, short axial length (AL), and smaller corneal diameter [1–3]. Recent investigations that have incorporated improved technology have added additional novel factors to this growing list. Using anterior segment optical coherence tomography (AS-OCT), Aung T and coworkers [4,5] found that smaller anterior chamber width (ACW), anterior chamber area (ACA), and anterior chamber volume (ACV); increased iris thickness, area, and curvature; changes in iris volume during dilation; and larger lens vaults were associated with PACD. On the other hand, our previous series of studies [6–10] using enhanced depth imaging optical coherence tomography (EDI-OCT) indicated that increased choroidal thickness (CT) plays an important role in the pathogenesis of PACD. Until recently, however, few studies have attempted concurrent measurement of the anterior and posterior ocular biometric characteristics or evaluation of the relationship between them.

The present study used AS-OCT and swept-source optical coherence tomography (SS-OCT; this procedure allows for the most accurate measurements of posterior segments currently possible) with the aim of providing concurrent normative values of the anterior and posterior ocular biometric characteristics and confirming the existence of a relationship between iris and choroid characteristics in healthy Chinese subjects. Moreover, gender deference of ocular biometry in healthy subjects and the relationships between biometric parameters with age were also investigated, which aims to explain why higher prevalence of angle closure rates are in the female gender and in aging people. Understanding the normal baseline of anterior and posterior ocular biometric parameters may aid in elucidating the pathophysiology of PACD and guide us toward more effective clinical detection.

## Methods

### Subject Recruitment

This cross-sectional study recruited 148 patients consecutively between January 2014 and June 2014 in accordance with the inclusion and exclusion criteria. All subjects were from the Chinese Han population. This study was approved by the Ethical Review Committee of the Zhongshan Ophthalmic Center. All participants received a detailed explanation about the study and signed an informed consent form, in accordance with the principles embodied in the Declaration of Helsinki.

All healthy subjects were recruited from employees and their families at the Zhongshan Ophthalmic Center, Sun Yat-Sen University, Guangzhou, China. Subjects were over 18 years old and had clear ocular media, normal visual field testing results, and no history of IOP exceeding 21 mmHg. Exclusion criteria were current ocular disease, previous ocular surgery, high myopia or hyperopia (spherical equivalent refractive error (RE) greater than + 6 or −6 diopters), clinically relevant opacities of the optic media, low-quality images due to unstable fixation, or severe cataract.

All subjects underwent a thorough ophthalmic evaluation, which included slit lamp biomicroscopy, IOP measurement (applanation tonometry), fundus examination, visual field text (SITA standard algorithm with a 24–2 test pattern; Humphrey Visual Field Analyzer II, Carl Zeiss Meditec, Dublin, California, USA), a RE examination using an auto refractometer (KR-8900 version 1.07, Topcon Corporation, Tokyo, Japan), and axial length measurements by partial optical coherence interferometry (IOLMaster; Carl Zeiss Meditec, Inc.).

### AS-OCT Measurements

**Fig 1:**
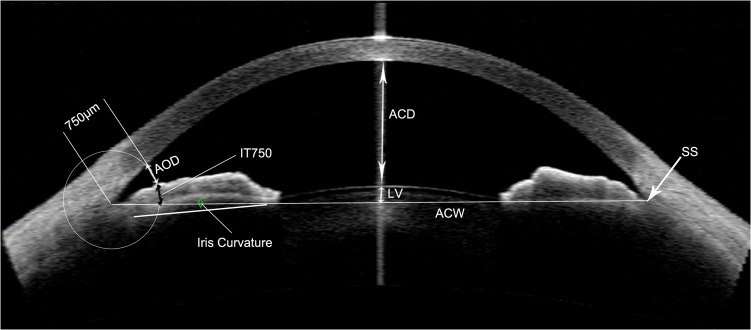
AS-OCT measurement. AS-OCT image showing the measurements of scleral spur (SS), ACD, ACW, LV, AOD750, IT750, and iris curvature.

Anterior chamber parameters were measured as described in previous studies [11,12]. AS-OCT (Visante OCT; Carl Zeiss Meditec, Dublin, California, USA) was measured under dark room conditions (0 lux) by a single operator. The scans were centered on the pupil to obtain a single cross-sectional horizontal scan (nasal-temporal angles at 0°–180°). During the examination, the examiner adjusted the noise and saturation and optimized the polarization to obtain the best quality image. Several scans were obtained for each subject, and the best image, with no motion or image artifacts by the eyelids, was selected. The images were then processed using the Zhongshan Angle Assessment Program (ZAAP, Guangzhou, China)[13]. The only operation performed on each image was to determine the location of the 2 scleral spurs. The software then automatically calculated the various anterior chamber parameters. The following parameters, as illustrated in Fig. 1, were measured: cornea thickness, ACD, ACW, ACA, ACV, pupil diameter (PD), angle opening distance at 750 μm from the scleral spur (AOD750), trabecular–iris space area at 750 μm from the scleral spur (TISA750), angle recess area (ARA), iris thickness at 750 μm from the scleral spur (IT750), iris curvature (ICURV), iris area (IAREA), and lens vault (LV).

### SS-OCT Measurements

After AS-OCT measurements, images of the macular region were obtained using the SS-OCT instrument (DRI OCT-1; Topcon, Tokyo, Japan). The light source of this SS-OCT system is a wavelength tunable laser centered at 1050 nm with an approximate 100-nm tuning range, yielding an 8-μm axial resolution in tissue. The device has been described in more detail elsewhere [14].

A 3-dimensional (3D) imaging scan protocol was used for evaluation of the macular region. The 3D imaging data set was acquired with a 6×6-mm raster scan centered on the fovea and composed of 256 B-scans, each consisting of 256 A-scans (total of 65,536 axial scans/volume). The scan protocol was repeated 3 times consecutively on the same visit. The participant and device were repositioned after each scan. Measurements of both eyes of each study participant were obtained through undilated pupils. Images included in the analysis had to have a quality score of 45 (of 160) or more, according to the manufacturer’s recommendation. All examinations were performed in the morning around 10 AM, to reduce the effects of diurnal variations. The frequency and effect of image artifacts were of interest in the present study. As a previous study described [15], the following sources of artifacts were of concern: motion artifact, signal loss resulting from blinking, and segmentation failure. The image artifacts were excluded, and the number of scans with an artifact and the type of the artifact were recorded.

**Fig 2:**
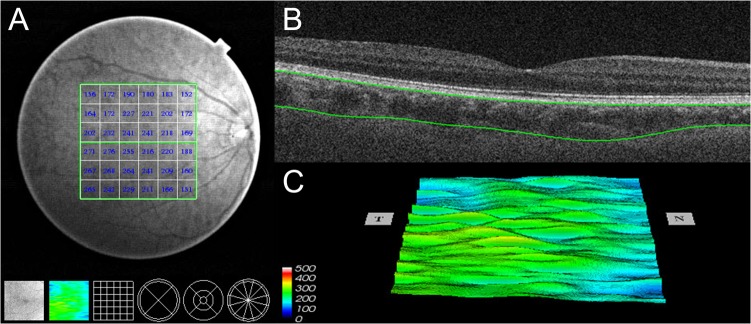
SS-OCT measurement. SS-OCT image showing the measurements of choroidal thickness. (A) Choroidal thickness map of the 6×6 mm area centered on the fovea was created. The mean choroidal thickness was obtained for each sector. (B) Automatic placement of the chorioscleral border made by the automatic built-in software in one of the B-scan images of the 3D data set. (C) Choroidal topographic map of the 6×6 mm area.

Choroidal and retinal thickness measurements were performed using built-in software (9.12.003.04). A 6×6 mm thickness map of 5 layers (inner limiting membrane (ILM), ganglion cell layer (GCL), ganglion cell complex (GCC), retina, choroid) was created by automated segmentation. The 6×6 grid was used for the thickness map (Fig. 2), and the mean regional thicknesses of the 5 layers were calculated for the 36 sectors of the grid.

### Statistical Analysis

The data were processed and analyzed statistically using SPSS (Version 13.0; SPSS, Chicago, IL). The intraclass correlation coefficient (ICC) was used to calculate the degree of reproducibility of SS-OCT measurement. Demographic data, as well as clinical measurements, were tabulated for all participants and by gender. The significance of gender differences was determined using an independent sample t-test. Pearson correlation analysis was performed to evaluate the relationships between ASOCT/SSOCT parameters with age. Univariate linear regression and multivariable linear regression were used to identify potential participant characteristics that were associated with iris thickness, iris area, and choroidal thickness. The association between choroidal thickness and iris parameters was calculated. To reach more precise conclusion, if negative results were found, power calculation were performed using PASS (Version 11.0; NCSS, LLC). For all the tests, p<0.05 was considered to be significant.

## Results

This study recruited a total of 296 eyes in 148 subjects. For AS-OCT measurements, 14 eyes were excluded because of poor-quality images, software delineation error, or indeterminate scleral spur. For SS-OCT measurements, after quality control and exclusion of scans with artifacts, 12 eyes were excluded because of low-quality images or artifacts. Therefore, complete data sets for both OCT measurement types were obtained for 270 eyes.

The reproducibility of SS-OCT measurement was measured in the first 50 included eyes. The ICCs for the mean ILM, GCL, GCC, retina, and choroidal thickness between two observers on the same visit were 0.97, 0.85, 0.84, 0.93, and 0.96, respectively (all p<0.01).

The subjects included 100 females and 46 males, with a mean (± standard deviation) age of 46.0 ± 14.4 years (range: 20–73 years). The mean IOP was 16.2 ± 2.68 mmHg. No significant differences were noted in age, IOP, or SE between female and male subjects. The mean axial length was 23.77 ± 1.02 mm. As expected, AL was significantly shorter in females than in males (p < 0.001). The demographic and clinical data are summarized in Table 1.

**Table 1:** Clinical characteristics of the study subjects.

Characteristic	Overall	Gender			Correlation with age	
		Female	Male	P Value*	r†	P Value†
No. of patients (No. of eyes)						
Total	148(270)	101(185)	47(85)	-	-	-
<30 y	20(40)	11(22)	9(18)	-	-	-
30–39 y	20(36)	10(18)	10(18)	-	-	-
40–49 y	28(51)	21(39)	7(12)	-	-	-
50–59 y	52(94)	40(73)	12(21)	-	-	-
>60 y	28(49)	19(33)	9(16)	-	-	-
Age, y	46.0(14.4)	47.1(13.7)	44.0(15.6)	0.199	-	-
IOP, mmHg	16.2(2.68)	15.5(4.03)	15.2(4.88)	0.279	-	-
Al, mm	23.77(1.02)	23.58(1.00)	24.15(0.95)	**<0.001**	-	-
SE, D	−0.20(2.18)	−0.03(2.10)	−0.52(2.32)	0.091	-	-
**ASOCT-anterior segment parameters**						
Corneal thickness, μm	566.8(30.3)	566.8 (30.7)	566.8 (29.7)	0.995	−0.031	0.639
ACD, mm	2.75(0.35)	2.69(0.34)	2.86(0.34)	**0.001**	−0.552	**<0.001**
ACW, mm	11.58(0.37)	11.51(0.36)	11.73(0.34)	**<0.001**	−0.207	**0.002**
ACA, mm^2^	21.25(3.88)	20.54(3.78)	22.70(3.70)	**<0.001**	−0.568	**<0.001**
ACV, mm^3^	142.5(33.7)	135.9(32.5)	156.2(32.1)	**<0.001**	−0.556	**<0.001**
PD, mm	4.69(0.88)	4.65(0.81)	4.77(1.01)	0.333	−0.336	**<0.001**
**ASOCT-mean anterior chamber angle parameters**						
AOD750, mm	0.41(0.21)	0.37(0.20)	0.48(0.21)	**<0.001**	−0.593	**<0.001**
TISA750, mm^2^	0.20(0.10)	0.19(0.09)	0.24(0.10)	**<0.001**	−0.524	**<0.001**
ARA, mm^2^	0.22(0.10)	0.20(0.10)	0.26(0.10)	**<0.001**	−0.481	**<0.001**
**ASOCT-iris and lens parameters**						
IT750, mm	0.46(0.08)	0.46(0.08)	0.45(0.07)	0.737	0.110	0.097
IAREA, mm^2^	1.49(0.24)	1.50(0.25)	1.47(0.23)	0.366	0.337	**<0.001**
ICURV, mm	0.23(0.14)	0.25(0.12)	0.19(0.16)	**0.003**	0.545	**<0.001**
LV, μm	326.7(299.4)	359.0(273.9)	260.0(338.6)	**0.019**	0.652	**<0.001**
**SSOCT parameters**						
ILM, μm	39.4(9.41)	39.8(9.50)	38.4(9.19)	0.279	−0.085	0.196
GCL,μm	73.1(23.0)	74.5(27.4)	70.2(5.68)	0.171	0.128	0.052
GCC, μm	111.3(15.9)	111.4(12.2)	111.2(22.1)	0.938	0.004	0.955
RNFL, μm	275.9(16.1)	275.7(16.6)	276.3(14.9)	0.760	−0.008	0.901
CT, μm	252.9(84.1)	240.5(72.8)	280.4(100.0)	**<0.001**	−0.311	**<0.001**

* P Value: Significance of differences between female and male: 2-samples independent t-test.

† r/P value: Pearson correlation between ASOCT/SSOCT parameters with age in all subjects.

Data are expressed as the mean (SD)

IOP = intraocular pressure; AL = axial length; SE = Spherical equivalent; D = diopter; ACD = anterior chamber depth; ACW = anterior chamber width; ACA = anterior chamber area; ACV = anterior chamber volume; PD = pupil diameter; AOD750 = angle opening distance at 750 μm from the scleral spur; TISA750 = trabecular–iris space area at 750 μm from the scleral spur; ARA = anterior chamber area; IT750 = anterior chamber volume; IAREA = iris area; ICURV = iris curvature; LV = lens vault; SD = standard deviation

### AS-OCT measurement parameters

Parameters measured by AS-OCT are shown in Table 1. Compared with males, the females had smaller anterior segment parameters (ACD, p = 0.001; ACW, p<0.001; ACA, p<0.001; ACV, p<0.001), narrower anterior chamber angle parameters (AOD750, p<0.001; TISA750, p<0.001; ARA, p<0.001), higher ICURV (p = 0.003) and larger LV (p = 0.019). The anterior segment parameters (ACD, ACW, ACA, ACV, PD), and anterior chamber angle parameters (AOD750, TISA750, and ARA) were negatively correlated with age (ALL p<0.01), while IAREA, ICURV, and LV were positively correlated with age (ALL p<0.001).

### SS-OCT measurement parameters

Parameters measured by SS-OCT are shown in Table 1. No significant differences were found in ILM, GCL, GCC, or retina thickness between females and males. CT was significantly smaller in females than in males (p<0.001). No significant correlation was found between ILM, GCL, GCC, and retina thickness with age, but CT was negatively correlated with age (p<0.001).

### Relationship between choroidal thickness and iris parameters

Uni- and multivariate linear regression analyses results (Table 2) showed the associated baseline factors for iris thickness, iris area, and choroidal thickness. The univariate regression analysis revealed a significant association for IT750 with ACW and PD (all p<0.05), while IAREA was associated with age, ACD, and PD (all p<0.05), and CT was associated with age, gender, AL, and SE (all p<0.05). Multivariate regression analysis showed similar results (IT750 associated factors were age, ACW, and PD; IAREA associated factors were age, ACW, and PD; and CT associated factors were age, gender, and AL; all p<0.05).

**Table 2:** Uni- and multivariate linear regression for associated baseline factors of iris thickness, iris area and choroidal thickness.

	univariate		multivariate	
	β* [95% CI]	P value*	β† [95% CI]	P value†
**IT750**				
age	0.001 [0.000,0.002]	0.097	0.001 [0.000,0.002]	**0.016**
gender	−0.004[−0.027,0.019]	0.737	-	**-**
AL	−0.006[−0.017,0.004]	0.230	-	**-**
ACD	0.000[−0.030,0.031]	0.984	-	**-**
ACW	−0.029[−0.058,−0.001]	**0.043**	−0.040[−0.070,−0.009]	**0.011**
PD	0.016[0.004,0.028]	**0.009**	0.027[0.013,0.040]	**<0.001**
**IAREA**				
age	0.007 [0.004,0.009]	**<0.001**	0.003 [0.001,0.005]	**0.003**
gender	−0.032[−0.101,0.037]	0.366	-	**-**
AL	−0.030[−0.061,0.002]	0.062	-	**-**
ACD	−0.133[−0.224,0.043]	**0.004**	-	**-**
ACW	−0.005[−0.092,0.082]	0.913	0.087[0.014, 0.160]	**0.020**
PD	−0.179[−0.207,−0.151]	**<0.001**	−0.167[−0.199,−0.136]	**<0.001**
**CT**				
age	−1.90 [−2.66,−1.14]	**<0.001**	−1.71 [−2.86,−0.56]	**0.004**
gender	39.92 [18.24,61.60]	**<0.001**	47.88 [19.82,75.95]	**0.001**
IOP	−1.12 [−4.45,2.19]	0.505	-	**-**
AL	−10.65[−20.31,−1.00]	**0.031**	−38.68[−54.31,−23.06]	**<0.001**
SE	6.75[0.99,12.52]	**0.022**	-	-
ACD	23.51[−8.80,55.83]	0.153	-	-
LV	−0.01[−0.05,0.02]	0.501	-	-

* β/P value: regression coefficient and P values of the independent variables in the univariate linear regression model;

† β/P value: regression coefficient and P values of the independent variables in the multiple linear regression model. Insignificant variables were not present in multivariate regressions;

In regression models, female was coded as 1 and male as 2 for gender;

95% CI: 95% confidence interval.

The relationship between CT and iris parameters is shown in Table 3. Univariate regression analysis revealed that increased CT was significantly associated with smaller IAREA (p = 0.026). However, this apparent association between CT and IAREA was abolished after adjusting for potential influence factors (including age, gender, AL, ACW, and PD).

**Table 3:** Uni- and multivariate linear regression analysis of the association between choroidal thickness and iris parameters.

	Unadjusted		Adjusted*	
	β [95% CI]	P value	β [95% CI]	P value
IT750	−46.01[−186.6,94.61]	0.520	−56.77[−186.4,72.91]	0.389
IAREA	−51.88[−97.64,−6.12]	**0.026**	−41.26[−94.76,−12.23]	**0.130**

*Adjusted for age, gender, AL, ACW, PD

## Discussion

The development of newer imaging modalities such AS-OCT has allowed researchers to capture the entire anterior segment in a single image for a more precise assessment of angle, iris, and lens parameters. On the other hand, the use of high-penetration SS-OCT allows researchers a more precise analysis of the posterior segment of the eye than was possible with previous OCT technology. The reproducibility of automated SS-OCT measurements in the present study was good, in accordance with the results found in a previous study.[15] The capability for precise acquisition of images of the anterior and posterior segment of the eye with concurrent AS-OCT and SS-OCT will lead to a better understanding of the anatomical characteristics of the eye and to the identification of additional anatomical risk factors that contribute to the pathogenesis of ocular diseases.

In the present study, we found that, when compared with male eyes, female eyes had narrower anterior biometric parameters that presented with smaller anterior segment parameters (including ACD, ACW, ACA, and ACV), narrower anterior chamber angle parameters (including AOD750, TISA750, ARA), higher ICURV, and larger LV. These anterior ocular biometric parameters were also correlated with increasing age. These gender differences and the aging trend may be helpful in explaining the higher prevalence of PACD rates in females, and in aging people [16,17]. Our posterior segment observation revealed a thicker CT in males than in females, and a significant negative correlation with age. This biometric feature in our series of subjects was similar to previous results obtained with Chinese subjects.[18,19]

Recent studies have emphasized the role of the choroid and iris in PACD [6–10,20] and have reported that increased iris thickness and area, as well as thicker CT, are associated with PACD risk. Iris thickness and area have been considered as one of the anatomic features in the “non–pupil block” mechanism for PACD.[20,21] The thick iris occupies a larger proportion of the anterior chamber volume in the angle recess area. With dilation of the pupil, the thickening of the peripheral iris will become even more pronounced and the iris may come into contact with the trabecular meshwork, thereby dramatically increasing the risk of angle closure.[22] Chinese subjects are also believed to have thicker irises than westerners, which would further increase the susceptibility to angle closure. In addition, according to Quigley et al.[23], thicker CT, coincident with intraocular volume increases, could cause an increase in vitreous cavity pressure. The pressure difference between the vitreous cavity and the posterior chamber would cause the lens to move forward, which would worsen the pupil block.

In the present study, our aim was to obtain a better understanding of the pathogenesis of PACD, primarily by focusing on the biometric features of the iris and choroid. Regression analysis indicated that iris thickness and area were significantly associated with age, ACW, and PD, while CT was associated with age, gender, and AL. However, if we are to obtain a better understanding of the ocular biometry, we must move beyond analysis of anatomic factors alone and view the situation as a whole.

Both the iris and choroid originate from the uvea, which is the vascular middle layer of the eye, so we attempted to find a relationship between these two structures. Univariate regression analysis showed that an increased CT was significantly associated with a smaller iris area, yet the underlying mechanism for this was unclear. Both the iris and choroid are full of blood vessels derived from the same artery system (ophthalmic artery), so we hypothesized that blood flow may play an important role in this association. A smaller iris area might increase the blood flow resistance of the long posterior ciliary artery (LPCA), which might cause an increase in the blood flow of the short posterior ciliary artery (SPCA), since both LPCA and SPCA originate from the ophthalmic artery. Increased blood flow would then cause choroid thickening [24].

However, after adjusting for potential influence factors (including age, gender, AL, ACW, and PD), no association between CT and iris area was found. The suggestion of association between the iris and choroid seen in our study is intriguing, but further longitudinal studies are needed to evaluate the dynamic changes between iris and choroid, especially in angle closure patients, before any firm conclusions can be drawn.

Some potential limitations in our study should be mentioned. First, we investigated the relationship between iris and choroid only in healthy subjects, whereas a more meaningful result might be obtained by investigating this relationship in angle closure patients, because an association has been found between both the iris and choroid and PACD risk. Second, we only measured anatomic ocular parameters in static images. The influence of dynamic factors, such as changes in iris area with pupil dilation [20] or choroidal changes induced by accommodation [25], should not be ignored. However, evaluations of dynamic factors are limited by the difficult nature of the procedures for image capture and measurement. New algorithms that include dynamic components are needed for these types of measurement. Third, the cross-sectional nature of the study precluded establishment of temporal or causal relationships. A prospective longitudinal study is needed to address the cause and effect relationship between the dynamic change of iris and choroid.

## Conclusion

Despite its limitations, this study is the first to use AS-OCT and SS-OCT for concurrent measurement of anterior and posterior biometric parameters, and it presents some valuable findings. Compared with male eyes, female eyes had narrower anterior biometric parameters that were correlated with increasing age, a finding that could explain the higher prevalence of PACD rates in the female gender and in aging people. Our attempt to find a relationship between the biometric features of the iris and choroid indicated that an increased CT might be associated with a smaller iris area. However, this needs to be investigated in future studies before a firm conclusion can be made.
